# Partition dependence in financial aid distribution to income categories

**DOI:** 10.1371/journal.pone.0231135

**Published:** 2020-04-17

**Authors:** Chenmu Xing, Katherine Williams, Jamie Hom, Meghana Kandlur, Praise Owoyemi, Joanna Paul, Ray Alexander, Elizabeth Shackney, Hilary Barth

**Affiliations:** 1 Department of Psychology, Wesleyan University, Middletown, Connecticut, United States of America; 2 Department of Addiction Studies, Psychology, and Social Work, Minot State University, Minot, North Dakota, United States of America; Middlesex University, UNITED KINGDOM

## Abstract

When allocating resources, people often diversify across categories even when those categories are arbitrary, such that allocations differ when identical sets of options are partitioned differently (“partition dependence”). The first goal of the present work (Experiment 1) was to replicate an experiment by Fox and colleagues in which graduate students exhibited partition dependence when asked how university financial aid should be allocated across arbitrarily partitioned income brackets. Our sample consisted of community members at a liberal arts college where financial aid practices have been recent topics of debate. Because stronger intrinsic preferences can reduce partition dependence, these participants might display little partition dependence with financial aid allocations. Alternatively, a demonstration of strong partition dependence in this population would emphasize the robustness of the effect. The second goal was to extend a “high transparency” modification to the present task context (Experiment 2) in which participants were shown both possible income partitions and randomly assigned themselves to one, to determine whether partition dependence in this paradigm would be reduced by revealing the study design (and the arbitrariness of income categories). Participants demonstrated clear partition dependence in both experiments. Results demonstrate the robustness of partition dependence in this context.

## Introduction

Grouping the same set of options in different ways will alter the way people choose from or distribute resources to different options [1]. A growing body of research has been devoted to examining how decisions are influenced by the structure of available options. When adults are asked to choose from a menu of options, they often employ a diversification heuristic and vary their selections across the options that are provided [2–4]. People may diversify their choices over both individual options and over arbitrary or subjective groupings of options, and because of this, different groupings can lead to different choices: a phenomenon called *partition dependence* [1, 5]. Partition dependence has been demonstrated across a wide range of contexts in adults (e.g., [1, 6]), and, recently, in a study of resource allocation by children [7].

Studies of partition dependence in adults have made use of a variety of paradigms, including experimental and nonexperimental field studies that involve donations to charity, wine and food selections, and prediction market prices [1, 2, 8]. For example, Fox, Ratner, and Lieb [1] carried out several experimental studies that highlight how our perception of category options alters the way in which we distribute choices or resources. In one study, students made choices about how to allocate a fixed number of free lunches over a future time period (an academic year) that could be partitioned in different ways, and their allocations were influenced by the way in which the academic year was subdivided [1]. Specifically, the Fall and the Spring semesters can each break down into two halves (Term I & II in the Fall; Term III & IV in the Spring). Individuals who were given a partition that presented two subdivided Fall terms individually along with a full Spring term (i.e., I-II-Spring) chose to assign more free lunches in the Fall, compared to those who were given a Fall-Spring partition or a Fall-III-IV partition that presented two subdivided Spring terms as individual units along with a full Fall term. In a study of hypothetical donations to charities, participants who were asked to give their proposed donations among the five charity fund options presented as five distinct partitions (one international fund and four local funds) gave an average of 21% (about one fifth) to the international fund. However, when given the same list of international versus local charities with the latter listed individually as four smaller subcategories under a superordinate “local” category, participants allocated on average 55% (about a half) to the international fund [1]. A vignette-based study showed that health-care providers made prescription decisions under the influence of the arbitrary partitioning of the available treatment options, providing evidence for partition dependence not only in multi-item but also in single-item choices, and not only in quantitative but also in qualitative decisions. Primary care providers in this study were less likely to choose more aggressive treatment options when they were grouped together, compared to when they were individually listed [9].

Because many adult studies make use of relatively complex verbal instructions and concepts that are not available to young children, the range of possible paradigms that can be used with child participants is more limited. However, Reichelson and colleagues [7] recently developed a novel paradigm based on the structure of previous adult studies that would allow for testing of partition dependence in children’s choices during a simple resource allocation game. In this game, children (preschoolers to elementary school age) were asked to distribute food tokens to different types of zoo animals, categorized in different manners. Children’s allocations revealed strong partition dependence effects at all ages tested, and younger children showed greater effects than did older children [7]. Thus, this recent evidence shows that partition dependent decisions are made early in life and continue throughout the lifespan, at least under some conditions.

Partition dependence is probably not a unitary phenomenon resulting from a single cognitive mechanism. However, it has been argued that a variety of partition dependent behaviors resulting from situations that differ considerably in their surface characteristics can be understood as part of a more general cognitive phenomenon in the following manner. When people are tasked with allocating some limited resource (e.g., money, probabilities, etc.) over a set of possibilities (such as consumer products, recipients, etc.), their final allocations usually represent some compromise between intrinsic preferences for one or more options and general tendencies toward equal allocation across options [6]. For example, people often feel motivated to be fair when allocating resources, and they may take into account relative need by applying need-based fairness norms [10]. A study of university financial aid allocations illustrates this idea [1]. Graduate students were asked to allocate hypothetical financial aid resources across several income brackets. Participants were assigned to either a low-income partition condition, in which they were presented with income categories equal to $75,000/year or more and five lower options (the lower options being $15,000 or less, $15,001-$30,000, $30,001-$45,000, $45,001-$60,000, and $60,001-$75,000 per year), or a high-income partition condition, in which they were presented with one category of $75,000/year or less and five higher options (the higher options being $75,001-$85,000, $85,001-$100,000, $100,001-$120,000, and $120,001-$145,000, and greater than $145,000 per year). (These are the ranges listed in the main text of the original study; a slightly different set of ranges is listed in a table in that study.) The income partitions were explicitly described to participants as being arbitrary in this study. Although participants in both conditions allocated more aid to the categories with lower income, presumably reflecting a need-based fairness norm, their allocations were also influenced greatly by the income bracket partitions—reflecting the tendency to distribute allocations equally among available options, even when those options are arbitrary. Specifically, participants allocated much more of the funding to families with incomes at $75,000 or less in the low-income partition condition and much less to such families in the high-income partition condition.

Considerable research on partition dependence in adults’ decisions has revealed that the phenomenon is generally robust and wide-ranging. Patterns of decision-making that are consistent with partition dependence have been observed, as described earlier, under many different conditions. However, it is less common, to our knowledge, for findings from particular partition dependence paradigms to have undergone multiple demonstrations of replicability. Indeed, at least one specific paradigm that has been reported to yield partition dependence has failed to do so in a series of replication studies. Simple choices of candies from an arrangement of bowls were reported to exhibit partition dependence in one study [1], but no such effect was found in three recent replication attempts, including one with very closely matched methods [11]. None of the established factors known to reduce partition dependence appeared to play a role in this study (e.g., expertise or greater intrinsic preferences; [1, 12]), showing that physical partitions like those utilized in the candy bowl study did not reliably influence consumer choice [11]. Simple selections for consumption from menus of physically partitioned options, therefore, do not necessarily produce partition dependent behavior as had previously been reported.

It is important to identify and understand the conditions that do reliably elicit partition dependent behavior in decision makers. One goal of the present work, therefore, is to replicate the findings of a previous study that reported partition dependent behavior. The consumer choice studies in which partition dependence findings were not replicated [11] made use of a relatively simple, straightforward selection task. For the present work, we aimed to move from consumer choice to an allocation task, while continuing to use a relatively simple, straightforward paradigm to attempt to replicate findings of partition dependence in adults’ decisions. Toward this end, we chose the financial aid allocation study described above [1] for a direct replication attempt in Experiment 1, using the same basic design. However, we also chose to carry out the study under somewhat adverse conditions for partition dependence in one sense: the participant population for the present study (members of the community of Wesleyan University, an undergraduate-focused liberal arts college in Middletown, CT, USA) is one that might well be expected to exhibit less partition dependence for financial aid related decisions. Individuals with stronger intrinsic preferences are sometimes less susceptible to partition dependent decisions about the objects of their preferences [1, 12]. In 2013, Wesleyan University removed its need-blind admissions policy, which led to debates and demonstrations on campus by many students who were concerned with how need-based admissions decisions would affect the socioeconomic diversity of the campus [13]. As a result, financial aid practices and the role of need in admissions became prominent topics of debate within the Wesleyan community. Wesleyan students and community members therefore might be expected to possess particularly strong preferences about how financial aid decisions should be made. We hypothesized that these participants might exhibit little, or reduced, partition dependence for decisions about financial aid allocations: such a finding would not be taken as evidence against the existence of partition dependence, of course, because it would be consistent with prior findings of the mitigating effects of preferences. Alternatively, a positive finding of partition dependence under these stringent conditions would provide particular evidence of the robustness of the effect in the context of this paradigm.

A second goal of the present work was to introduce a modified version of the financial aid allocation paradigm (Experiment 2) to test whether in the present task context, participants would demonstrate partition dependence even when provided with a clear demonstration of the arbitrariness of the presented categories (see [8, 12]). In previous work with the financial aid paradigm [1] participants were verbally and explicitly instructed that the presented income partitions were arbitrary, but they were only introduced to one of the two possible partitions of income range, as in Experiment 1. In Experiment 2, on the other hand, we showed participants that the categories they were given were arbitrary by revealing both possible partitions of income range. In this “high transparency” version of the task, participants saw both the low-income and high-income bracket ranges, and assigned themselves to one range based on the parity of the last digit of their student ID number. The fact that different participants would be presented with different income categories was therefore demonstrated openly to all participants in Experiment 2 (because it would presumably be obvious that some participants would have even student ID numbers, while others would have odd numbers). Related designs have been used in some previous studies. In an experiment that used prediction market pricing tasks to elicit participants’ perceived probability of the event value of a series of events, participants were assigned to one of two partitions but were told about both [8, Exp.1]. Such an instruction was argued to be particularly important for this type of paradigm because traders tend to process a given partition by perceiving it as a rational market-based design rather than an arbitrary grouping [8]. A similar approach has been used in other experiments [12, Exps.4 & 5]. In these experiments, participants randomly assigned themselves to specific combinations of market events for probability judgment and partition types based on the last digit of their telephone number. In the present work, we extended this design approach to the financial aid allocation paradigm. We hypothesized that participants exposed to this modified task might exhibit little or even no reliable evidence of partition dependence. Materials and data are available at https://osf.io/xa98k/, DOI 10.17605/OSF.IO/XA98K.

## Experiment 1

In Experiment 1, participants were asked to allocate financial aid to income categories that were differently partitioned across conditions: some participants experienced the upper end of the presented income range partitioned into more categories, and others experienced the same range but with the lower end partitioned into more categories.

### Method

#### Participants

Participants were recruited on the campus of Wesleyan University. They were primarily Wesleyan undergraduates with some non-student community members also included. One hundred and twenty-one adults (*M*_age_ = 20.80, *SD* = 1.50, range = 18.27–29.90, 79 females; 3 participants chose not to report gender) were included in the final analysis. One of these did not provide requested information about financial aid status, resulting in a total of 120 participants for any potential analysis involving financial aid status. Twenty-three additional participants were excluded from data analysis due to noncompletion (*n* = 1, 0% of aid was allocated); allocating more or less than 100 percent of the budget (*n* = 17); response of “yes” or missing response to the question “Have you participated in a similar study at Wesleyan before?” (*n* = 3); or lack of Wesleyan community member status (*n* = 2).

#### Procedure

Participants completed the task in student centers on campus and received either candy or a pencil for their participation. Participants were randomly assigned by the experimenter to one of the two income partitions (low, *n* = 62, or high, *n* = 59), and they were only aware of the existence of that income partition. The study used the basic experimental design of [1, Experiment 1]. After informed written consent was obtained, the participant was told that the Wesleyan University financial aid office would spend $42.6 million on need-based financial aid in the upcoming school year. They were then asked to respond to this hypothetical scenario:

“Suppose that you were to advise the financial aid office on how they should distribute next year’s budget among entering freshmen who apply for financial aid. Specifically, you are asked to indicate what **percentage** of the budget you would allocate to aid applicants whose family household incomes fall in various ranges. Please indicate the percentage of the budget that you recommend should be allocated to that group of students. Feel free to indicate 0% or 100% for any of the categories below.”

The last sentence in the hypothetical scenario above, i.e., “Feel free to indicate 0% or 100% for any of the categories,” also included in the original study of Fox and colleagues, was intended to reduce any demand effects such as a potential implication that the presented categories were meaningful. After reading the hypothetical scenario, participants were reminded that the percentages needed to add up to 100. The design was a direct replication of the Fox, Ratner, and Lieb study [1]. Participants saw a single table of six income intervals. Those randomly assigned to the low-income partition condition saw intervals ranging from “$15,000 or less” to “more than $75,000” (see Table 1 for all intervals). Those randomly assigned to the high-income partition condition saw intervals ranging from “$75,000 or less” to “more than $145,000” (see Table 1). Participants were asked to allocate 100% of available financial aid across the six different income ranges by writing down the percentage that should be allocated to each interval. After participants distributed financial aid percentages across the income ranges, in any condition, participants were asked to write down whether they were current recipients of financial aid and what they thought the study was about. There were no additional measures or conditions that are not reported in this manuscript. Both experiments presented here received approval from the Psychology Department Ethics Committee at Wesleyan University. All participants gave informed written consent prior to participating.

**Table 1:** Mean percentage of financial aid allocated to each income range by each condition in Experiment 1.

**Low-Income Partition**	**Low Transparency**
**Income (x $1,000)**	**Mean % Allocated**
≤ 15	32.6
15–30	22.6
30–45	17.5
45–60	13.2
60–75	8.8
> 75	5.3
**High-Income Partition**	**Low Transparency**
**Income (x $1,000)**	**Mean % Allocated**
≤ 75	49.4
75–85	22.5
85–100	13.7
100–120	7.5
120–145	4.7
> 145	2.2

### Results and discussion

The proportion of participants receiving financial aid was similar across the conditions (low-income partition: 55.7%, high-income partition: 62.7%), *χ*^2^ (1, N = 120) = .604, *p* = .437.

**Fig 1:**
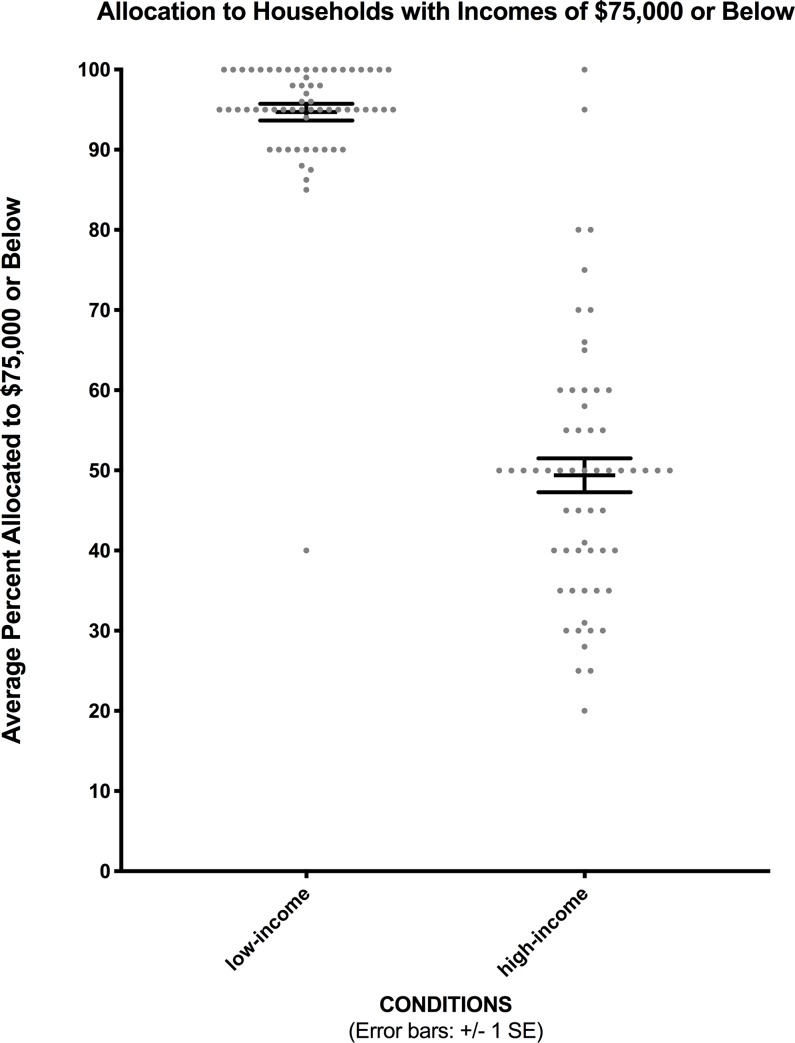
Financial aid allocations for both conditions in Experiment 1. Data points show individual participants’ allocations.

Table 1 gives the mean percentage of financial aid allocated to each income range for each condition in Experiment 1. Basic findings of the original study were replicated. Like Fox and colleagues [1], we found that participants in both the low and high income partition conditions preferred to give more money to families with lower income levels: the lowest income category received the greatest mean percentage, and increasing income levels were given monotonically decreased mean percentages. We also replicated the finding that participants chose to spread allocations over the categories they were given, though they were told that the categories were arbitrary and the instructions did not prevent allocations of 100% or 0% to any income interval. Our focus is specifically on the percentage of financial aid allocated to households with incomes of $75,000 or less: evidence of partition dependence would be present if more funds were allocated to this income level in the low-income partition condition than in the high-income partition condition [1]. Participants in the low-income partition condition distributed considerably more financial aid (*M* = 94.7%; *SD* = 8.2%) to households with incomes of $75,000 or less than did those in the high-income partition condition (*M* = 49.4%; *SD* = 16.3%), *F* (1, 119) = 379.871, *p* < .001, *η*^2^ = .76, as Fig 1 shows. This is consistent with the findings of [1], with very similar values resulting (95.9% allocated to such households in the low-income partition condition, vs. 47.7% in the high-income partition condition).

Many of our participants were current financial aid recipients (*N* = 71), suggesting that the participants in our study might have had particularly strong intrinsic preferences concerning financial aid decisions. To investigate whether participants currently receiving financial aid exhibited less partition dependence, a two-way ANOVA was conducted with partition type and student financial aid status (yes or no). Overall, participants receiving financial aid allocated a higher percentage of aid to households with incomes of $75,000 or less (adjusted means 73.9% vs. 69.0%), *F* (1, 116) = 4.358, *p* = .039, with a relatively small effect size, *η*_***p***_^2^ = .036. However, most importantly, we found no interaction between financial aid recipient status and income partition type, *F* (1, 116) = 0.825, *p* = .366. The main effect of income partition type remained strong: *F* (1, 116) = 379.369, *p* < .001, *η*_***p***_^2^ = .77. Even though current financial aid recipients did allocate more financial aid to households with incomes of $75,000 or less, the analyses did not provide evidence that financial aid status moderated the way income partitions influenced resource allocation in our study.

## Experiment 2

In Experiment 2, we asked whether participants would demonstrate partition dependence even when provided with a clear demonstration of the arbitrariness of the presented categories in a “high transparency” modification of the task. We did so by showing participants that the categories they were given were arbitrary by revealing both income partitioning possibilities. Participants saw both the low-income and high-income bracket ranges, and assigned themselves to one range based on the parity of the last digit of their student ID number.

### Method

#### Participants

Participants were recruited on the campus of Wesleyan University. They were primarily Wesleyan undergraduates with some non-student community members also included. Ninety-eight adults (*M*_age_ = 20.72, *SD* = 1.45, range = 18.13–26.47, 61 females; 3 participants chose not to report gender) were included in the final analysis. Nineteen additional participants were excluded from data analysis due to noncompletion (*n* = 1, 0% of aid was allocated); allocating more or less than 100 percent of the budget (*n* = 10); response of “yes” or missing response to the question “Have you participated in a similar study at Wesleyan before?” (*n* = 7); or having prior knowledge of the study’s purpose (*n* = 1).

#### Procedure

Participants completed the task in student centers on campus and received either candy or a pencil for their participation. Participants were presented with *both* the low-income partition table and the high-income partition table side-by-side (such that they observed both sets of income intervals). Participants were instructed to allocate 100% of available financial aid across one partition of the six different income ranges by randomly assigning themselves either to the low-income or high-income partition based on the last digit of their student identification number. Participants with an ID number ending in an odd number allocated financial aid for the low-income partition categories (*n* = 48) and those with an ID number ending in an even number allocated financial aid for the high-income partition categories (*n* = 50). Other than this difference, the design was the same as in Experiment 1.

As in Experiment 1, after participants distributed financial aid percentages across the income ranges, in any condition, participants were asked to write down whether they were current recipients of financial aid and what they thought the study was about. There were no additional measures or conditions that are not reported in this manuscript.

### Results and discussion

The proportion of participants receiving financial aid was similar across the conditions (low-income partition: 58.3%, high-income partition: 54.0%), *χ*^2^ (1, N = 98) = 0.187, *p* = .666.

Table 2 gives the mean percentage of financial aid allocated to each income range for each condition in Experiment 2. Again, basic findings of the original study were replicated. Participants in both the low- and high-income partition conditions preferred to give more money to families with lower income levels. They allocated the largest mean percentage for the lowest income category, and mean percentage allocated decreased monotonically with increasing income level. Participants chose to spread allocations over the available categories, though they were told that the categories were arbitrary and the instructions did not prevent allocations of 100% or 0% to any income interval. Notably, the present study finds the very same patterns in the high-transparency version of the task in which participants had the opportunity to observe both sets of categories (demonstrating their arbitrariness openly).

**Table 2:** Mean percentage of financial aid allocated to each income range by each condition in Experiment 2.

**Low-Income Partition**	**High Transparency**
**Income (x $1,000)**	**Mean % Allocated**
≤ 15	30.2
15–30	21.1
30–45	16.9
45–60	14.5
60–75	10.2
> 75	7.0
**High-Income Partition**	**High Transparency**
**Income (x $1,000)**	**Mean % Allocated**
≤ 75	51.7
75–85	19.7
85–100	12.6
100–120	7.4
120–145	6.2
> 145	2.4

**Fig 2:**
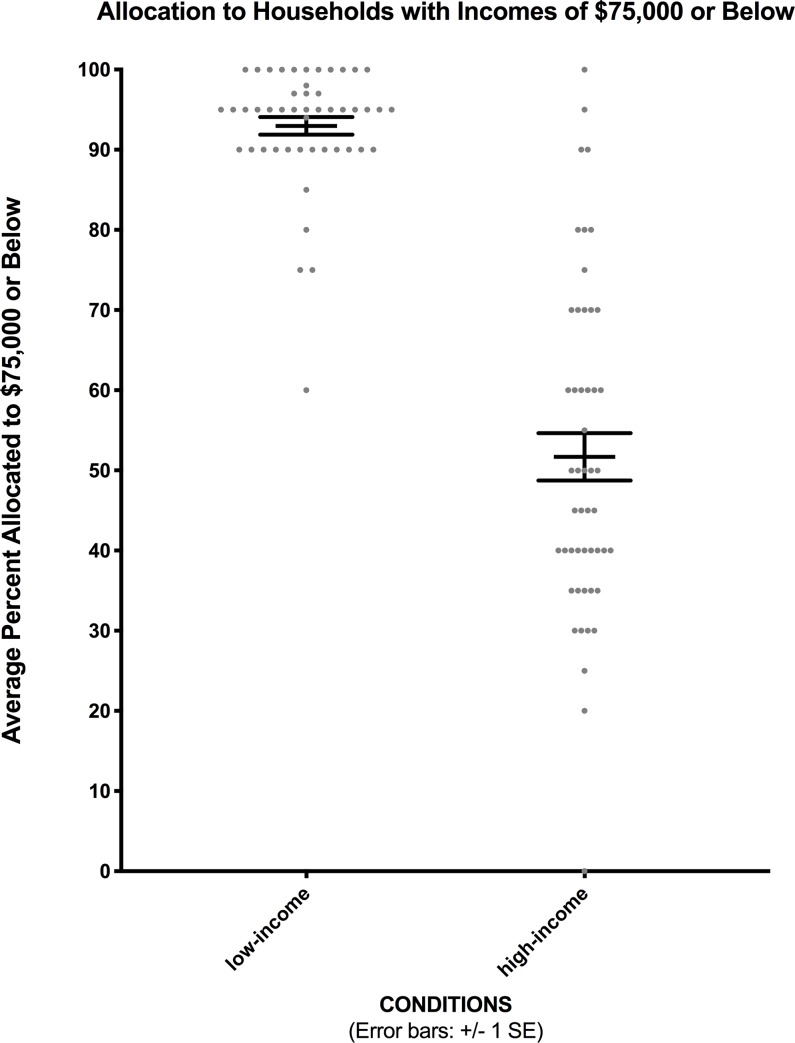
Financial aid allocations for both conditions in Experiment 2. Data points show individual participants’ allocations.

As in Experiment 1, we focused on the percentage of financial aid allocated to households with incomes of $75,000 or less: partition dependence would be observed if more funds were allocated to this income level in the low-income vs. high-income partition condition. In the high-transparency task of Experiment 2 (just as in the low-transparency task of Experiment 1), participants in the low-income partition condition distributed considerably more financial aid (*M* = 93.0%; *SD* = 7.7%) to households with incomes of $75,000 or less than did participants in the high-income partition condition (*M* = 51.7%; *SD* = 20.8%), *F* (1, 96) = 166.701, *p* < .001, with a large effect (*η*^2^ = .63), as Fig 2 shows.

Also as in Experiment 1, to investigate whether participants currently receiving financial aid exhibited less partition dependence, a two-way ANOVA was conducted with partition type and student financial aid status (yes or no, recipients *n* = 55). As in Experiment 1, participants receiving financial aid allocated a higher yet statistically comparable percentage of aid to households with incomes of $75,000 or less (adjusted means 74.1% vs. 70.1%), *F* (1, 94) = 1.533, *p* = .219. Most importantly, we found no interaction of financial aid recipient status with the income partition type in the high transparency condition groups, either, *F* (1, 94) = .012, *p* = .913. The main effect of partition type also remained strong here: *F* (1, 94) = 162.012, *p* < .001, *η*_***p***_^2^ = .63. Even though current financial aid recipients did allocate more financial aid to households with incomes of $75,000 or less, the analyses did not provide evidence that financial aid status moderated the way income partitions influenced resource allocation in our study. To compare, the partition effect observed in the low-transparency task (Experiment 1) was *F* (1, 119) = 379.871, *p* < .001, *η*^2^ = .76.

We also combined data across the low transparency task (Experiment 1) and the high transparency task (Experiment 2), with the caveat that direct comparisons across experiments should be made with caution (as participants were randomly assigned to conditions within each experiment, but not across experiments). A two-way ANOVA tested the main and interaction effects of partition type (low vs. high) and transparency type (low vs. high) on the percentages of financial aid allocated to households with incomes of $75,000 or less. There was a main effect of income partition type: participants assigned to the low-income partitions allocated a significantly higher average percentage of financial aid to such households than those assigned to the high-income partitions (adjusted means 93.8% vs. 50.5%), *F* (1, 215) = 501.657, *p* < .001, with a large effect size, *η*_***p***_^2^ = .70. On the other hand, there was no main effect of level of transparency (adjusted means 72.0% at low transparency vs. 72.3% at high transparency), *F* (1, 215) = .024, *p* = .877. There was no interaction, *F* (1, 215) = 1.082, *p* = .299. Participants in both tasks therefore exhibited robust partition dependence effects, and the degree of partition dependence observed was the same for both the low- and high-transparency tasks. Finally, we conducted the same analyses on a larger subset of the participants in Experiments 1 and 2, selected to include as many participants as possible and therefore including everyone who produced interpretable data (*N* = 250 participants, *M*_age_ = 20.76, *SD* = 1.50, range = 18.13–29.90, 157 females; 7 participants did not report gender), and the results remained unchanged.

## General discussion

The present work had two main goals: to replicate previously reported findings of partition dependence in a resource allocation task, and to extend this work by asking whether partition dependence would arise in a high-transparency version of the paradigm. This version has been previously used for some paradigms (e.g., prediction market choices) but not in the context of resource allocation, to our knowledge. In Experiment 1, we aimed to replicate an experiment reported by [1, Exp.1] in which adults were asked to determine how university financial aid should be allocated across different income brackets grouped into arbitrary partitions. We presented this task to a rather different sample: community members at a liberal arts college that has been the site of recent policy changes and heated debates regarding financial aid practices, many of whom are current financial aid recipients themselves. We reasoned that a demonstration of partition dependence in financial aid allocation within this community would provide strong evidence of the robustness of the effect in the context of this task, and indeed we did find evidence of partition dependence. Specifically, participants diversified their allocations of financial aid across the displayed arbitrary income brackets, as in [1], and although they did allocate more funds to households with less income, the amount allocated to households with incomes of $75,000 or less was dramatically changed by the arbitrary partitioning of the presented income categories across participants. When the low end of the income range (i.e., $75,000 or less) was partitioned into multiple income categories (the low-income partition condition), much more aid was allocated to these households. When the high end of the income range (i.e., more than $75,000) was partitioned into multiple income categories (the high-income partition condition), much less aid was allocated to these households.

Our second goal was to extend the financial aid allocation findings with an adaptation of the paradigm that made the arbitrariness of the presented income categories highly salient and transparent. In the replication study described above, each participant was only aware of one possible set of income categories; they were explicitly described as arbitrary, but participants were not explicitly made aware of other possible partitioning options. In a “high-transparency” version of the task presented in Experiment 2, participants were shown two possible income partitions and randomly assigned themselves to one, which they then used to make their financial aid allocations. Even under these conditions, with both possible sets of income categories revealed such that the arbitrariness of the income categories was made transparent, participants demonstrated clear and strong partition dependence: the amount allocated to households with incomes of $75,000 or less was again dramatically changed by the arbitrary partitioning of the presented income categories. Moreover, we did not find reliable evidence that partition dependence was reduced for participants who observed both possible sets of income categories. We also found that although current financial aid recipients (who made up a large part of our sample) did tend to allocate a slightly higher percentage of aid to families with less income, there was no evidence that they exhibited different partition dependence effects. A potential limitation of the present replication work is the relatively small sample size given some recent recommendations [14]. For future replication attempts, a sample size that is 2.5 times the original study sample size would be ideal.

These findings are important for at least two reasons. First, they support considerable previous work (e.g., [1, 6]) showing that individuals’ decisions are highly susceptible to simple partitioning effects such that marketers, policymakers, and other choice architects have broad opportunities to influence decisions simply by manipulating the presentation of available options (see also [15–16] for a review). Second, the current findings highlight the robustness of this particular type of partition dependence effect. Despite using experimental conditions and contexts that arguably decreased the likelihood that we would observe partition dependence in this study, we found strong evidence of partition dependence, with large effect sizes in both the high- and low-transparency versions of the task.

Numerous studies show that decisions of all kinds are heavily shaped by the arbitrary option partitioning that happens to be most accessible or available to the decision maker at the time of the decision. Although rich descriptions and characterizations of these phenomena are available, to our knowledge less is known about their psychological explanations–perhaps because the broad pattern of partition dependence is instantiated within many more specific domains that may involve a wide range of cognitive mechanisms. Some work has begun to explore psychological mechanisms that may be at work; for example, recent studies have explored the idea that partitions are interpreted as though they convey relevant information to participants [17]. Recent developmental work may also be able to place constraints on potential mechanisms: because there is evidence of partition dependence in at least some types of paradigms even in very young children, it cannot be generated (at least in these contexts) by mechanisms unavailable early in development [11]. Elucidating the potential mechanisms responsible for partition dependent decision making is a key topic for future research.

Existing research highlights a need for improved understanding of partition dependence and how this phenomenon could lead to improvements in how we make decisions, especially in situations that have material consequences on people’s opportunities and quality of life (e.g., consumer markets, health-care provision, and financial investment). The present study contributes to this literature by demonstrating that partition dependence effects are clearly present at least in resource allocation paradigms of this type, and further shows that such partition dependence effects are robust even in the face of attempts to render transparent the arbitrary nature of the presented allocation categories.
